# Dual 500-μs wide pulse neuromuscular electrical stimulation enhancing sensorimotor cortical excitability

**DOI:** 10.3389/fnhum.2025.1629003

**Published:** 2025-07-28

**Authors:** Yun Zhao, Yanying Yan, Xiaoling Zhang, Guanghui Xie, Renqaing Yang, Shuaidong Zou, Fengmei Gao, Wencheng Sun

**Affiliations:** School of Smart Health, Chongqing Polytechnic University of Electronic Technology, Chongqing, China

**Keywords:** neuromuscular electrical stimulation, variable-frequency trains, cortical excitability, afferent fibers, EEG

## Abstract

**Background:**

Neuromuscular electrical stimulation (NMES) is an effective tool to improve motor activation of patients with motor dysfunction. However, to enhance the cortical activities induced by NMES, the corresponding strategies should be carefully designed with optimal stimulation parameters. The aim of the present study is to investigate whether the pulse assignment with wide-pulse-based Variable Frequency Trains improves sensorimotor cortical excitability.

**Methods:**

A block-designed experiment was conducted with NMES delivering current to right biceps brachii muscle in nine healthy right-handed adults to evoke repetitive elbow flexion under similar kinetic parameters (*p* > 0.05). A new NMES pattern with the combination of wide-pulse and Variable Frequency Trains (wDFT, variable-frequency trains with 2-let frequency train) was set to compare with other NMES patterns, i.e., variable-frequency trains with narrow-pulse (nVFT, 8-let frequency train), constant-frequency trains with narrow-pulse (nCFT, one pulse), and CFT with wide-pulse (wCFT, one pulse). The excitability levels of sensorimotor regions were investigated based on beta event-related desynchronization (ERD) analysis.

**Results:**

Although evoking similar elbow flexion movements, variable-frequency trains (VFT) could induce stronger cortical activities than constant-frequency trains (CFT). Moreover, the sensorimotor cortex responded significantly more preferably to the dual 500 μs wide pulse VFT (wDFT) stimulation pattern (*p* < 0.05). In general, VFT induced higher amplitudes and descending slopes of beta ERD than CFT did during evoking elbow flexion movements, among which wDFT induced the highest beta ERD intensity and its descending slope (*p* < 0.05). In addition, the current efficiency of VFT to modulate sensorimotor cortical activities was higher than that of CFT pattern.

**Conclusion:**

The VFT pattern, especially dual 500 μs wide pulse VFT, could enhance sensorimotor cortical excitability, and the central neural activities improvements may attribute to the fact that more afferent fibers are effectively activated. Therefore, our findings indicated the high potential of utilizing DFT with wide pulses to optimize NMES applications in motor rehabilitation.

## 1 Introduction

Motor dysfunction after stroke is generally associated with a reduction of central nervous system (CNS) circuit excitability and a decreased cortical representation area corresponding to the affected limbs (Yu et al., 2024). Successful rehabilitation with partial/entire functional recovery may enhance brain activities that control spontaneous movements (Joy and Carmichael, 2021). Recently, neuromuscular electrical stimulation (NMES) has been considered as one sufficient intervention to induce high-level cortical excitability or rapid reorganization in sensorimotor cortices for patients with physical impairments (Carson and Buick, 2021; Zhang et al., 2023). Powell et al. (1999) have reported that NMES could increase recovery of wrist extension over standard care in hemiplegic patients, and it has been also demonstrated to induce neuroplasticity by increasing the baseline level of spinal excitability such that low levels of input result in voluntary motor function in the patients with spinal cord injury (Karamian et al., 2022). Brain imaging studies showed that the NMES-evoked movements induced reliable cortical excitability enhancement and plastic changes in specific neural regions (Tenberg et al., 2023; Insausti-Delgado et al., 2021). Further research on chronic stroke patients confirmed that changes in sensorimotor area are accompanied with effective hand recovery (Kimberley et al., 2004). It is clear that the NMES-induced activity change and adaptive reorganization in brain play an important role in human behavioral restorations following rehabilitation treatments. Researchers have attempted to improve the rehabilitation-mediated functional brain changes through optimizing the NMES patterns (Zhao et al., 2018; Obayashi and Saito, 2022; Ryoki et al., 2017). Parameters of the NMES patterns, such as stimulation intensity, frequency or pulse width were optimized to enhance the sensorimotor cortical excitability and subsequently impact the efficiency of rehabilitation (Makii et al., 2015; Wegrzyk et al., 2017; Jiang et al., 2019).

Usually, neuromuscular electrical stimulations directly activate motor axons beneath the electrodes to induce isometric contractions (i.e., efferent/peripheral pathway) (Bergquist et al., 2011). Also, the electric pulse can generate contractions through reflexive recruitments of motoneurons by depolarizing the sensory fibers and ascending afferent volleys that contribute to cortical activity regulation (i.e., afferent/central pathway) (Qu et al., 2020). Therefore, the neuroplasticity changes in the central nervous system driven by NMES may be substantially related to increasing sensory axons recruitments. The sensory fibers and motor axons were reported to have different electrical excitabilities depending upon their intrinsic properties including strength-duration time constant and rheobase (Veale et al., 1973; Kiernan et al., 2004). Accordingly, the pulse duration was considered as a major factor affecting the priority of sensory or motor axon recruitments in NMES (Wegrzyk et al., 2017). Some recent studies have reported the effect of stimulation intensity and stimulation frequency on brain activation patterns (Insausti-Delgado et al., 2021; Backes et al., 2000; Kampe et al., 2000), but few studies have investigated whether stimulation pulse width affects cortical activities. The brain imaging study has just reported that the modulation of wide pulses resulted in differently activated brain regions, that is, NMES with wide pulses (100 Hz-1 ms) resulted in lower deactivation in the secondary somatosensory cortex and precuneus, while the conventional pattern (25 Hz–0.05 ms) induced larger hyperactivated in the bilateral thalami and caudate nuclei during stimulating the triceps surae of healthy subjects (Wegrzyk et al., 2017). Herein, the present study wanted to determine whether the optimized NMES pattern with wide pulses could better enhance cortical activities.

Traditional NMES pattern is constant-frequency trains (CFT) which generally composes of successive narrow stimulus pulses (< 400 μs) and is applied at repetitive frequency of 15-40 Hz (Collins, 2007). This conventional CFT stimulations can artificially elicit muscle contractions to recruit motor units (MUs) resulting from motor neurons depolarization primarily through efferent/peripheral mechanism (Wegrzyk et al., 2017) and less sensory fiber activation through afferent/central pathway. The low priority of sensory axons recruitments in CFT stimulation is attributed to the differential sensitivity and antidromic collisions between sensory and motor fibers (Veale et al., 1973; Kiernan et al., 2004). Recently, wide duration pulses (pulse width of 0.5–1 ms) have been reported a tendency of preferably stimulating more sensory rather than motor axons, causing the resultant afferent inputs ascending through spine pathway to enhance cortical activities in the central nervous system circuit (Bergquist et al., 2011). Panizza et al. (1992) demonstrated that wider stimulus duration (0.5–1 ms) facilitated eliciting Hoffmann reflexes (H-reflexes) in healthy volunteers which reflected information of the sensory fiber excitation (Rongsawad and Ratanapinunchai, 2018). Lagerquist and Collins (2010) also observed that NMES with wide pulses applied to 15 healthy subjects evoked more afferent volley.

Compared to conventional CFT, Variable Frequency Trains (VFT) is an alternative stimulus pattern that can evoke muscle contractions in a more physiological manner (Deley et al., 2014; Karu et al., 1995; Doll et al., 2018; Carole et al., 2016). Each of those electrical pulses in VFT could only induce subthreshold depolarizations of axons, and different numbers of subthreshold depolarizations could be temporally summed to activate different axons with different activation thresholds, leading to recruiting MUs in asynchronous pattern (Carole et al., 2016). MUs recruitment in asynchronous pattern mainly resulted from directly activating motor axons (efferent pathway) and indirectly recruiting motoneurons in the spinal cord by activating sensory axons (afferent pathway) (Bergquist et al., 2011). Carole employed a special stimulation protocol of VFT that consisted of N high-frequency stimuli (called N-lets) with long inter-period intervals (Carole et al., 2016), and doublet-frequency train (DFT, *N* = 2) was acknowledged as the most successful train in force improvement (Karu et al., 1995). While VFT has been reported more profit to evoking muscle contraction compared to CFT, studies have not investigated the effect of VFT, especially DFT with wide pulses, on cortical activities. Herein, we hypothesized that the pulse assignment with wide-pulse-based VFT neuromuscular stimulation would better enhance sensorimotor cortical activities due to the activation of more sensory fibers during evoking elbow flexion movements of right upper limb. The purposes of the present study were to evaluate the effect of pulse assignment of NMES patterns on the neural activations in sensorimotor areas and investigate whether dual wide-pulses in doublets have an advantage in the sensorimotor cortical excitability improvement during evoking functional elbow flexion movements. We evaluated sensorimotor cortical excitability through quantifying the Beta ERD from EEG data from Electrode C3 located in left sensorimotor cortex with the corresponding ERD properties (amplitude and descending slope).

## 2 Methods

### 2.1 Subjects

Nine healthy right-handed subjects (three females and six males, 24 ± 2 years old) were recruited. All subjects were confirmed with no history of former cardiovascular disease, neurological disorders or orthopedic problems in arms. Subjects were also free of upper limb resistance training in the past 6 months and no NMES treatment experience before. This study was reviewed and approved by the Ethics Committee of Chongqing Polytechnic University of Electronic Technology (approval code: 001/2024), and written informed consent was obtained from every participant, in accordance with the Declaration of Helsinki.

### 2.2 Experimental protocol

**Figure 1:**
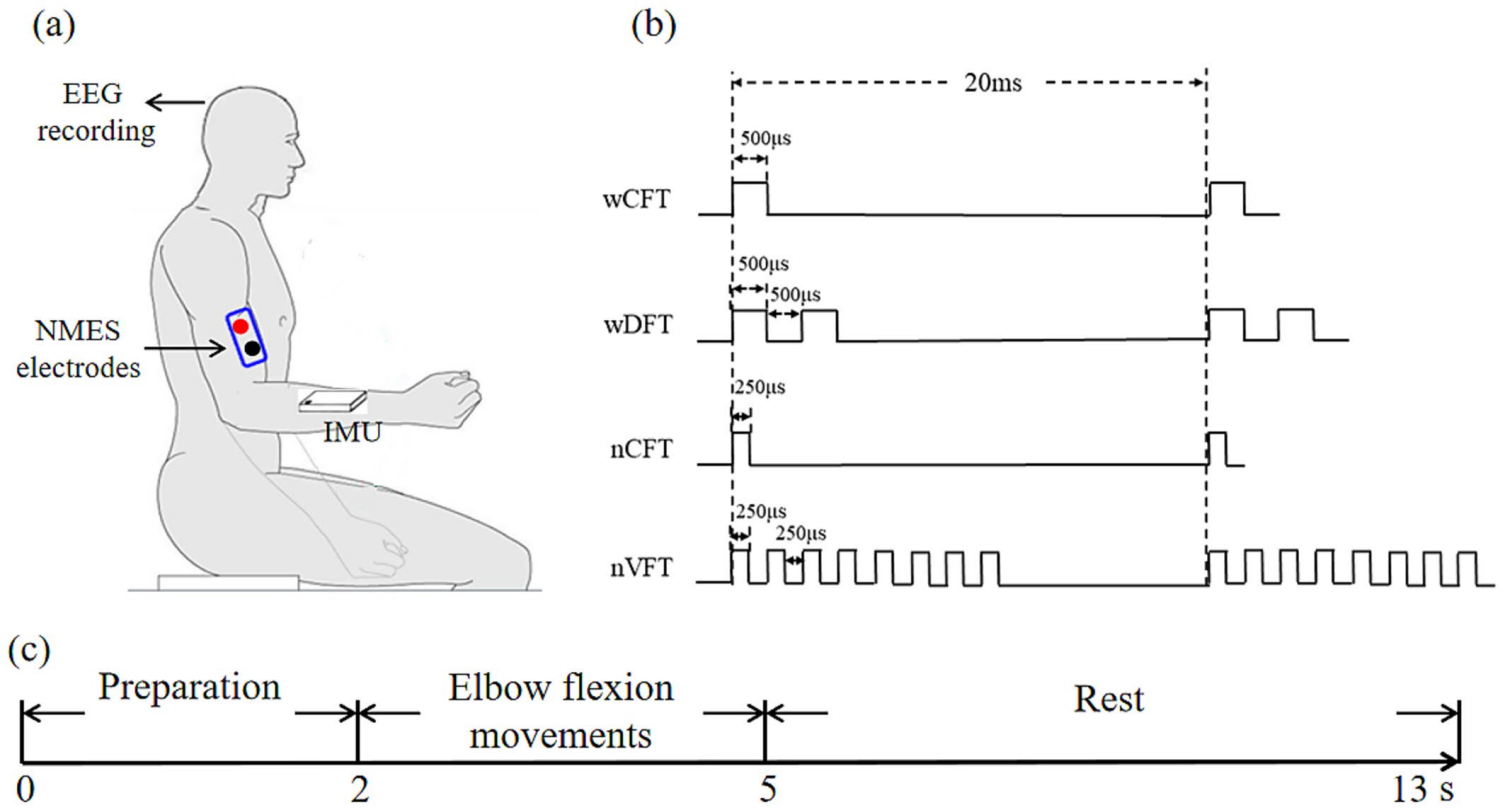
Experimental setup for sensorimotor cortex excitability assessment during NMES: **(a)** the experimental framework, **(b)** the designed NMES patterns for delivery, **(c)** the experimental paradigm of each trial.

The diagram of the experimental setup was illustrated in Figure 1a. The stimulation electrodes were placed on right biceps brachii muscle of all subjects to evoke the specific elbow flexion movements, and the stimulation current was delivered using Programmable Stimulator (Master-9, AMPI, Jerusalem, Israel) and isolated cables (ISO-Flex, AMPI, Jerusalem, Israel). An inertial measurement unit (IMU) (MPU6050, Vit motion, China) was placed on dorsal forearm of subjects to collect the kinematic data of elbow flexion movements. During the experiment, a multichannel wireless Electroencephalographic (EEG) acquisition system (STARSTIM-8, Neuroelectrics, Inc., Spain) was used to record EEG signals.

The stimulation protocol was consisted of four stimulation patterns (Figure 1b), i.e., VFT with narrow-pulse (nVFT, Variable Frequency Trains with 8-let frequency train), DFT with wide-pulse (wDFT, Variable Frequency Trains with 2-let frequency train), CFT with narrow-pulse (nCFT, one pulse), and CFT with wide-pulse (wCFT, one pulse). During the 3-s elbow flexion, electric pulse trains were delivered with a repetitive frequency of 50 Hz (burst frequency of pulse trains) with the pulse width of narrow pulse of 250 μs and the pulse width of wide pulse of 500 μs. The detail of stimulation parameters was illustrated in Table.1.

**Table 1:** The parameters of four types stimulation patterns.

Patterns	Pulse width	Burst frequency of pulse trains	Pulses per train
nVFT	250 μs	50 Hz	8
wDFT	500 μs	50 Hz	2
nCFT	250 μs	50 Hz	1
wCFT	500 μs	50 Hz	1

In each trial (13 s), subjects were required to keep their arm sagging naturally in a peaceful and relaxed state at the first 2 s for preparation; then a 3-s elbow flexion task (described in section 2.3) was performed and natural elbow extension followed to the initial position within an 8-s rest interval before next trial (Figure 1c). In addition, a 10-min rest interval was set between two elbow flexion tasks to avoid muscle fatigue. Experiments were carried out in a quiet room with room temperature kept at 26 ± 1°C.

### 2.3 Experimental tasks

Subjects paid two visits to the laboratory on 2 separate days with 1 day interval. The main purpose of the 1st visit was to determine the stimulating parameters, such as the maximum angle of elbow flexion. To determine the stimulation electrode position, the skin of all subjects was firstly prepared using an alcohol pad. Then we placed round hydrogel electrodes (3 cm in diameter) on the top of the right biceps brachii (BIC) muscle. Once determined the stimulation point, we marked and fixed the position by medical adhesive tape. Subjects were blind to these parameters during whole tasks.

The tasks were performed during the second visit. There were four tasks performed according to the order of nVFT-evoked elbow flexion movements, wDFT-evoked elbow flexion movements, nCFT-evoked elbow flexion movements, and wCFT-evoked elbow flexion movements. Each task was performed for 15 trials by every subject. Before the experiment, the surface skin on right biceps brachii muscle of all subjects was cleaned with medical alcohol so that the stimulation electrodes could have good contact with skin. Moreover, the stimulating electrodes were fixed with medical adhesive tape to avoid the displacement of the stimulation point. According to experiment instructions, the stimulator was turned on to evoke right biceps brachii muscle contraction to reach a specific angle position (50°) for a 3 s elbow flexion movement. Then the stimulator was turned off, and subjects put down their arm naturally back to initial position for a rest before next trial. To ensure all tasks to be performed in a certain movement pattern, a long handle protractor was used in all NMES-evoked tasks. During the experiment, subjects were required to close their eyes so as to have no visual access to the ongoing elbow flexion movement in order to avoid observation interferences.

### 2.4 Data recordings

EEG data were record with 8Ag/AgCl scalp electrodes (7 electrodes FC1, C3, CP1, FC2, C4, CP2, and Cz were installed in international 10/20 system, and the other one was placed below the right eye for recording the ocular artifact). The dual reference EARCLIP was placed on the right ear of subjects. The sampling rate was 500 Hz and band-pass filtering range was set from 2 to 40 Hz. Kinematic data were sampled at 100 Hz.

### 2.5 Data processing

All experimental data were processed in MATLAB2012b (MathWorks, Natick, MA) environment. EEG signals were firstly band-pass filtered with the frequency band of 2-40 Hz by the Butterworth filter in EEGLAB. Then, independent component analysis (ICA) algorithm in FastICA was adopted to remove the ocular artifact from EEG data (Delorme and Makeig, 2004). EEG signals were re-referenced to Electrode Cz. Before further analysis, EEG data were segmented in trials according to Figure 1c.

**Figure 2:**
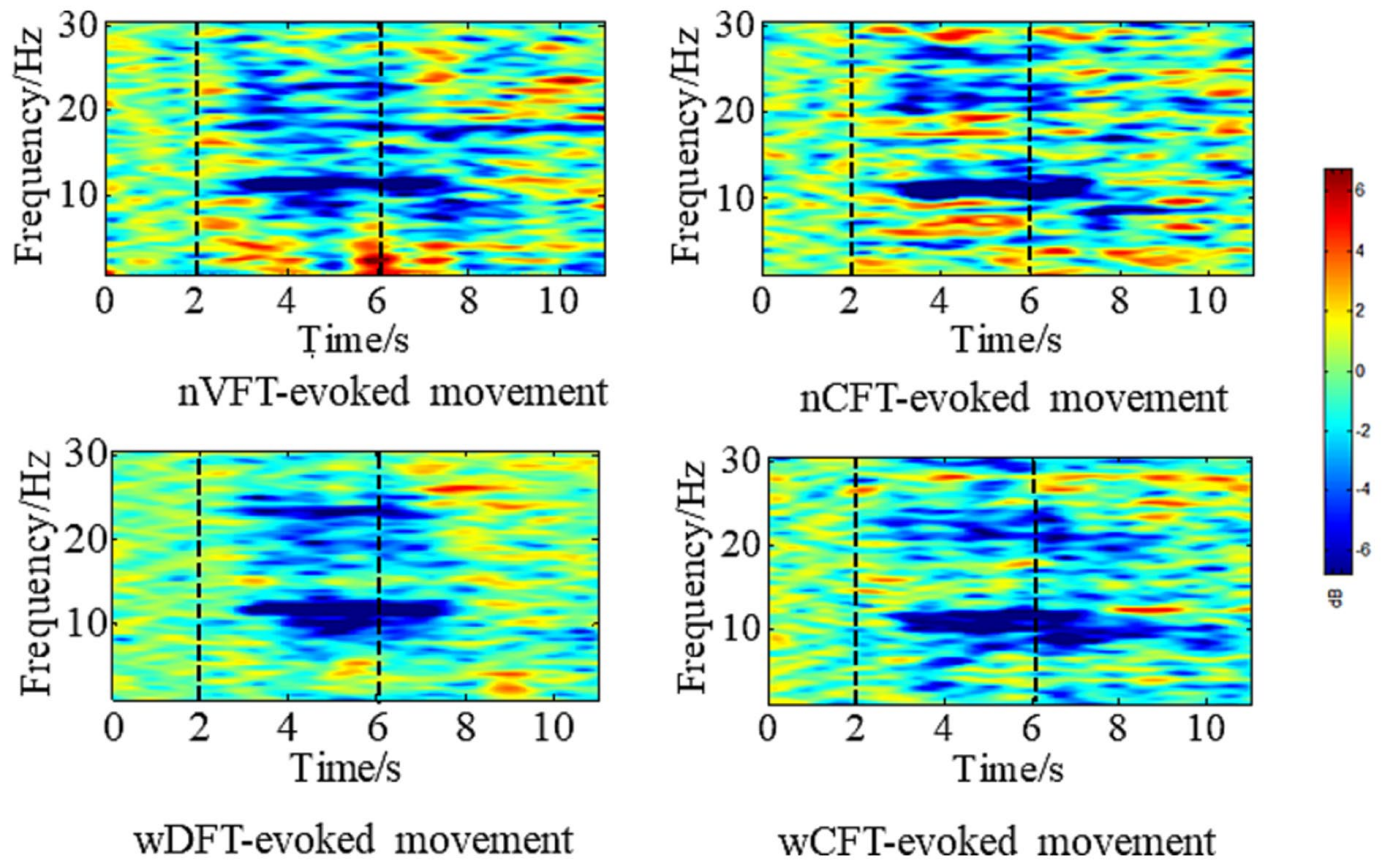
Averaged time-frequency maps at electrode position C3 during VFT-evoked elbow flexion movement, and CFT-evoked elbow flexion movement for all trials of one subject. Blue denotes ERD. The first vertical line denotes movement onset, the second one denotes task offset.

As Electrode C3 corresponds to primary sensorimotor cortex projecting to right upper limb, EEG data of Electrode C3 were analyzed trial by trial for all tasks. Based on the literatures (Müller et al., 2003; Wang et al., 2023), we analyzed the ERD of EEG in Beta rhythm (14–30 Hz) to evaluate the cortical excitability during NMES-evoked elbow flexion movements. Event-Related Spectral Perturbation (ERSP) was utilized to inspect the spectral power changes of EEG signals in time-frequency domain for ERD evaluation. The ERSP maps for Electrode C3 of all tasks were computed using Short-time Fourier transform (STFT) with a Hanning-tapered window applied in EEGLAB (Delorme and Makeig, 2004). Baseline-normalized ERSP values (dB) was calculated from 0 s to 13 s based upon the 2 s baseline of pre-onset period, as shown in Figure 2. The final ERSP values were averaged among all 15 trials in time-by-frequency plane for each task.

To investigate the amplitude of sensorimotor cortical oscillation during tasks, ERD value was extracted as follows in Equation 1:


(1) $$\mathrm{ER}D_{\text{value}}=\min_{14\leq K\ll 30}(\frac{1}{N}\sum_{f=K}^{K+2}\sum_{t=2}^{5}\text{ERSP}(f,t))\;$$

Where *N* is the number of time-frequency bins in a 2 Hz wide frequency band. Averaged ERSP values were extracted from 2 to 5 s after trial onset in the special time-frequency band by sliding a 2 Hz wide window from 14 Hz to 30 Hz, the minimum of which was selected as the ERD value. Since ERD is a negative value, the absolute ERD value $|\mathrm{ER}D_{\mathrm{abs}}=\mathrm{ER}D_{\text{value}}|$ was calculated for statistical analysis.

**Figure 3:**
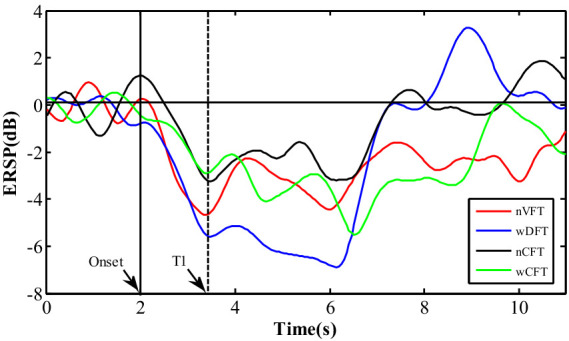
The temporal changes of ERSP values in corresponding frequency bin during the whole trial at contralateral sensorimotor cortex (C3): T1 is the moment when ERD value falls on a plateau segment.

Beta ERD changes in corresponding frequency bin, named as *ERSP_T_* (as shown in Figure 3), were used to investigate temporal characteristics of the sensorimotor cortical excitability. The descending slope *ERD_Slope_* of *ERSP_T_* against time was calculated from the movement onset to the achievement of *ERSP_T_* plateau as in Equation 2:


(2) $$\mathrm{ER}D_{\text{Slope}}=|\mathrm{ER}D_{T1}-\mathrm{ER}D_{T0}|/T1$$

Where T1 is the moment when ERD value falls to a plateau phase, and T0 is the moment of the movement onset, *ERD*_*T*1_ and *ERD*_*T*0_ are ERD values in the time at T1 and T0, respectively.

To further evaluate the stimulation efficiency under different electric pulse assignments, we calculated the neural activations (ERD) of per stimulation input (current and phase charge) as:


(3) $${\mathrm{ERD}}_{\text{current}}=\frac{\mathrm{ERD}}{I}$$

(4) $${\mathrm{ERD}}_{\text{charge}}=\frac{\mathrm{ERD}}{Q}\;$$

Where ERD is the beta ERD value obtained in Equation 1, and I is the stimulation intensity (current in mA) in Equation 3. *Q* is the phase charge (in μC) in Equation 4, the current-time integral of the pulses each burst (Doll et al., 2018).

Kinematic data were also segmented in trials according to Figure 1c. Angular curves of elbow flexion movements across all 15 trials were averaged task by task. Angular range and its corresponding movement duration were extracted to evaluate the movement performance in different tasks.

### 2.6 Statistical analysis

Kinematic parameters (angular range and duration with elbow flexion respectively) and EEG activities (*ERD_abs_* and *ERD_Slope_*) of all elbow flexion movements were analyzed with one-way repeated measures ANOVA. The elbow flexion movement was set as an independent factor. In addition, current intensity ( $I_{\text{value}}$ ) and current efficiency ( ${\mathrm{ERD}}_{\text{current}}$ ) were also analyzed by one-way repeated measures ANOVA with NMES patterns as an independent factor and the current intensity or current efficiency as a within-subject factor. The Least Significant Difference (LSD) correction was used to make the *post-hoc* pairwise comparisons. Furthermore, phase charge (*Q*) and its efficiency ( ${\mathrm{ERD}}_{\text{charge}}$ ) were compared between nVFT and wDFT by a paired *t*-test. Statistical analyses were performed using SPSS 22 (SPSS Inc., Chicago, Illinois), and the significance level was set at *p* < 0.05 for all procedures.

## 3 Results

### 3.1 Elbow flexion movements evoked by all NMES patterns

**Figure 4:**
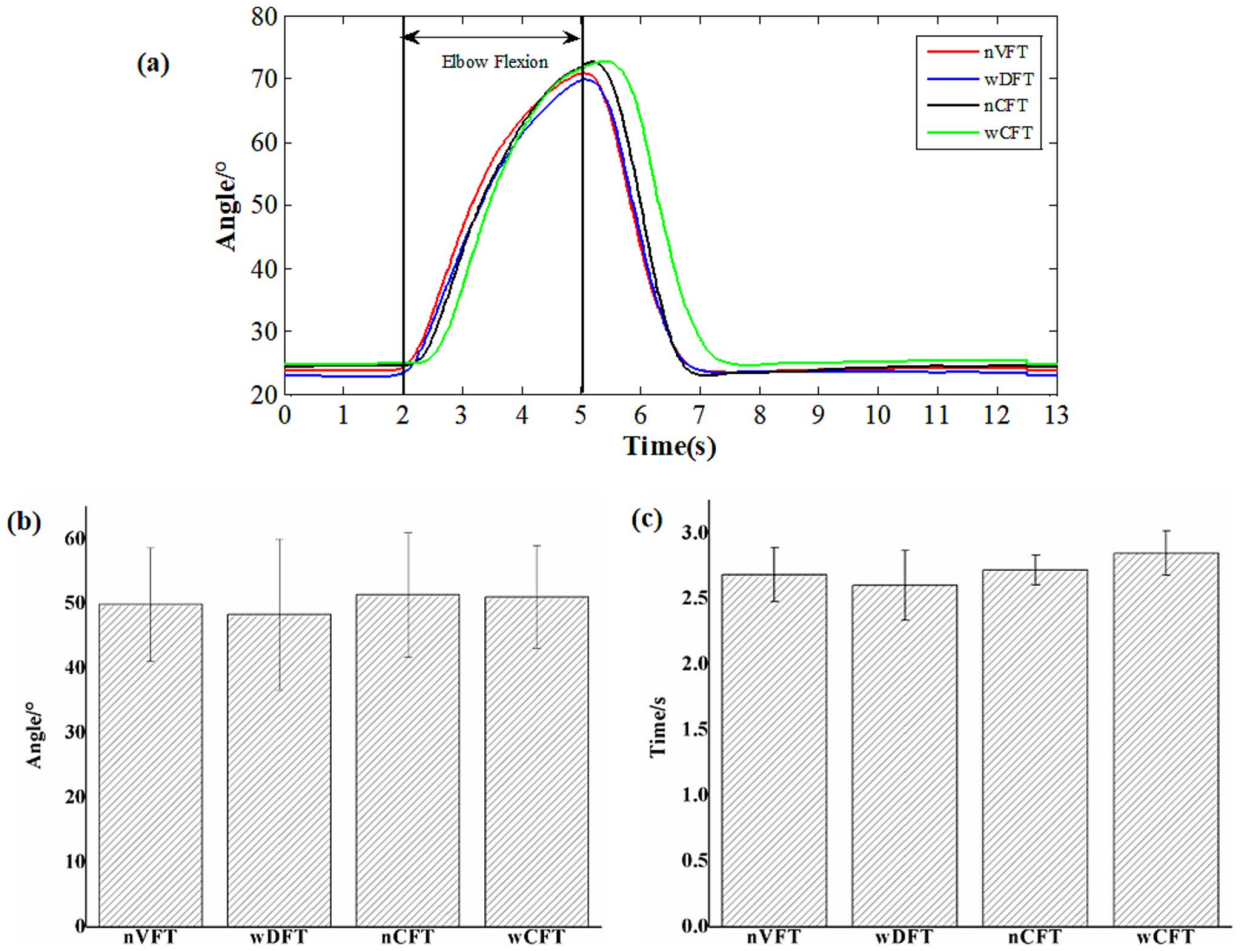
Kinematic measurements of all elbow flexion tasks: **(a)** angle curves, **(b)** the statistical results of angular ranges, **(c)** the statistical results of the durations of all NMES-evoked elbow flexion movements.

Figures 4a,b showed angular curves and the statistical results of angular ranges for all NMES-evoked movements, respectively. As the elbow flexion movements were performed, all angular curves began to increase at 2 s and reached their peak at 5 s, followed by the kinematic curves for the arm back to the initial position which were not analyzed in this study. The statistical results of angular ranges showed no significant difference (*p* > 0.05) among different NMES-evoked tasks (nVFT: 49.87 ± 8.75°, wDFT: 48.30 ± 11.67°, nCFT: 51.35 ± 9.64°, wCFT: 51.02 ± 7.92°). Meanwhile, the durations from initial movement onset to the moment of the peak angle of all evoked movements were statistically calculated (Figure 4c), and the results showed also insignificantly different duration (*p* > 0.05) among all the elbow flexion tasks (nVFT: 2.68 ± 0.20s, wDFT: 2.60 ± 0.27 s, nCFT: 2.72 ± 0.11 s, wCFT: 2.85 ± 0.17 s respectively). Combining the above results with the parameters set for this study that the stimulation duration of each trial was 3 s and the initial movement and the ending positions of elbow flexion were fixed for all tasks, it could be concluded that all NMES patterns evoked similar elbow flexion movements.

### 3.2 VFT vs. CFT on cortical activities

**Figure 5:**
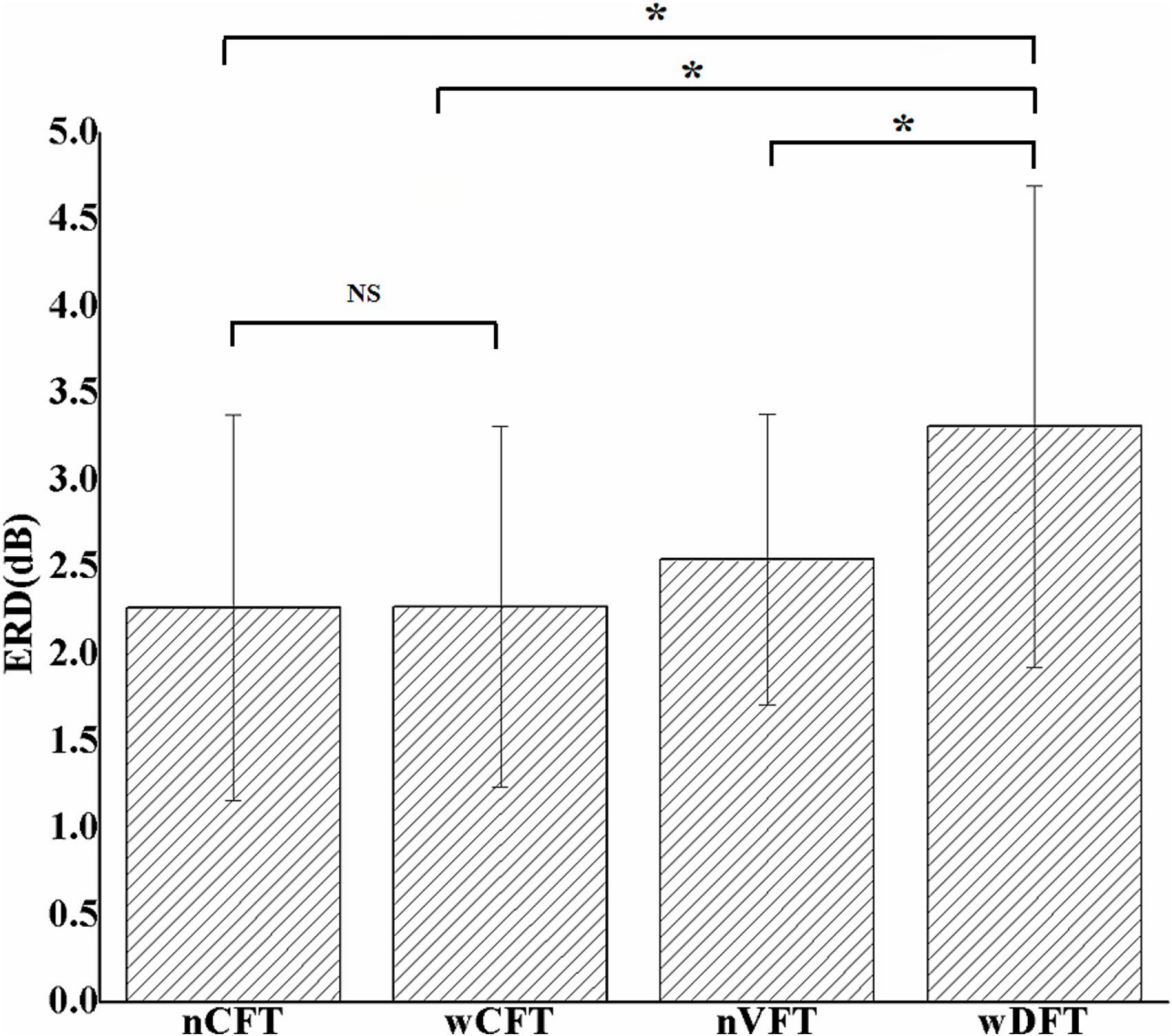
The averaged absolute beta ERD values at contralateral sensorimotor cortex (C3) among all elbow flexion tasks. Stars indicate significance: ∗indicates *p* < 0.05, and NS indicates no significance.

The time-frequency information of EEG data at electrode C3 was analyzed for all elbow flexion tasks, and the ERD patterns in beta rhythm were extracted after movement onset (Figure 2). Although all the tasks showed the movements with similar kinematic performance, the induced sensorimotor cortical activities were significantly affected by stimulation patterns [*F*_(3, 34)_ = 7.781, *p* < 0.005]. As shown in Figure 5, stronger cortical excitability was induced via VFT rather than CFT. The beta ERD values (3.31 ± 1.3 dB) during wDFT-evoked elbow flexion tasks were significantly higher than those (2.27 ± 1.10 dB) during wCFT-evoked elbow flexion tasks (*p* < 0.05). nVFT-evoked elbow flexion movement also induced higher beta ERD values (2.55 + 0.83 dB) than nCFT-evoked tasks (2.26 ± 1.11 dB) did, although the difference (*p* > 0.05) was insignificant. It can be observed that wDFT-evoked elbow flexion induced highest ERD intensity among all NMES-evoked tasks (*p* < 0.05). In contrast, beta ERD value did not significantly respond to pulse duration fluctuations in CFT, as no significant difference was observed between wCFT and conventional nCFT according to the beta *ERD_abs_* metric (*p* > 0.05).

**Figure 6:**
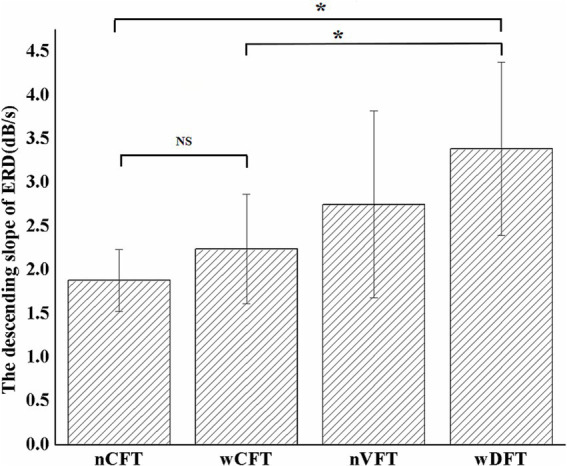
The averaged descending slope of beta ERD values at contralateral sensorimotor cortex (C3) among all elbow flexion tasks. Stars indicate significance: ∗indicates *p* < 0.05, and NS indicates no significance.

To further investigate the cortical activity modulation effects of different stimulation patterns, the descending slope *ERD_slope_* of beta ERD values at contralateral sensorimotor cortex (C3) was calculated (Figure 6), and it was significantly affected by stimulation patterns [*F*_(3, 34)_ = 3.462, *p* < 0.05]. The *ERD_slope_* responding to wDFT (3.39 ± 0.99 dB/s) was significantly greater (*p* < 0.05) than that responding to both wCFT (2.34 ± 0.63 dB/s) and nCFT (1.88 ± 0.36 dB/s) patterns, indicating that beta ERD in sensorimotor area could reach the value in the time of T1 faster during wDFT-evoked elbow flexion. Moreover, the *ERD_slope_* induced by nVFT (2.75 ± 1.1 dB/s) was also higher than that of both conventional nCFT and wCFT though the difference was not significant (*p* > 0.05).

### 3.3 wDFT vs. nVFT on phase charge efficiency

**Figure 7:**
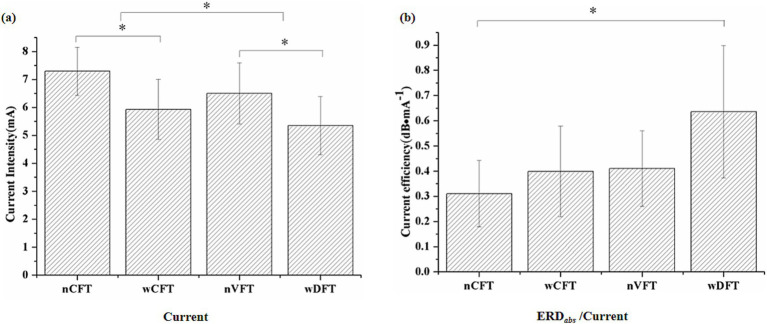
Current intensity **(a)** and its efficiency **(b)** among all four NMES patterns. Stars indicate significance: ^∗^indicates *p* < 0.05.

Although evoking similar elbow flexion movements, four NMES patterns delivered different current intensities [*F*_(3, 24)_ = 3.874, *p* < 0.05]. As shown in Figure 7a, with the same pulse duration, current intensity of VFT was significantly lower than CFT. wDFT (5.35 ± 1.04 mA) required significant lower current intensity (*p* < 0.05) than wCFT (5.93 ± 1.07) did. nVFT (6.5 ± 1.09 mA) required significant lower current intensity (*p* < 0.05) than nCFT (7.29 ± 0.86 mA) did. Furthermore, we explored whether there were differences in the current efficiency when NMES patterns induced sensorimotor cortical excitability. The current efficiency of all stimulation patterns was calculated according to Equation 3. It can be seen from Figure 7b that the current efficiency of wDFT (0.64 ± 0.26 dB/s) was significantly higher (*p* < 0.05) than that of both nCFT (0.31 ± 0.13 dB/s) and wCFT (0.39 ± 0.18 s). The current efficiency of wDFT was same as to that of nVFT (0.41 ± 0.15 dB/s). The current efficiency of nVFT was also higher than that of the two CFT patterns, although there was no significant difference (*p* > 0.05). Therefore, the current efficiency of VFT to modulate sensorimotor cortical activities was better than that of CFT pattern.

**Figure 8:**
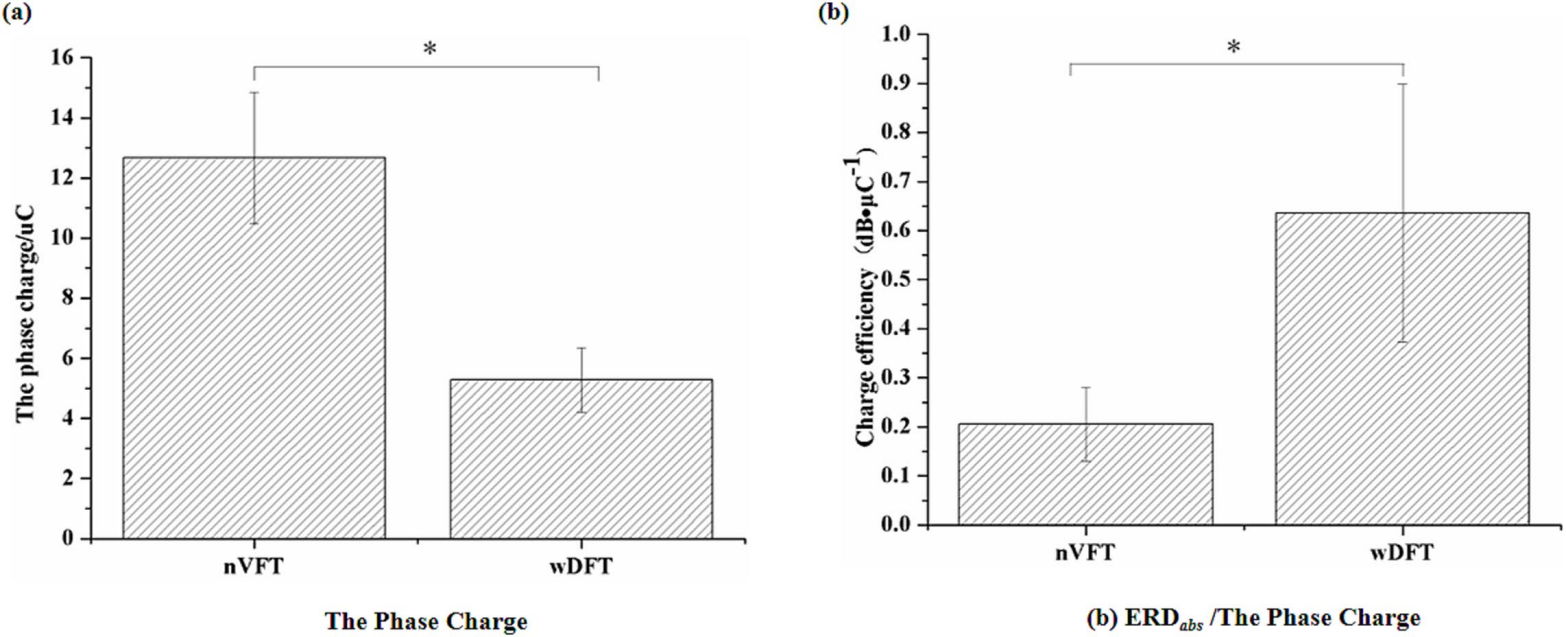
The phase charge **(a)** and its efficiency **(b)** between wDFT and nVFT. Stars indicate significance: ^∗^indicates *p* < 0.05.

Due to different stimulation intensities, pulse widths and durations of each train, the phase charge was used to synthetically characterize the pulse delivery pattern of VFT, and then the phase charge efficiencies (beta ERD amplitudes induced by unit phase charge) to activate cortical excitability were compared between two VFT patterns (nVFT vs. wDFT). It could be observed in Figure 8 that with similar evoked elbow movements, wDFT required significantly less phase charges than nVFT (*p* < 0.05). As mentioned above, the beta ERD amplitudes induced by wDFT was significantly higher than that of nVFT (*p* < 0.05). Therefore, the phase charge efficiency of the beta ERD intensity induced by wDFT-evoked elbow flexion movements was significantly higher than that by nVFT-evoked movements (*p* < 0.05), as shown in Figure 8b. Those results indicated that the wide-pulse VFT had better stimulation efficiency of modulating sensorimotor cortical activities.

## 4 Discussion

The present study investigated the impact of dual wide-pulses in doublets on sensorimotor cortical excitability improvement by combining the wide pulse with Variable Frequency Trains. Beta ERD of EEG signals from Electrode C3 located in left sensorimotor cortex was utilized to evaluate the activation level in cortex region during elbow flexion tasks (Wang et al., 2023; Tacchino et al., 2016; Wang et al., 2024). VFT stimulation could induce stronger sensorimotor cortical excitability than CFT stimulation, especially, the DFT with the dual 500 μs wide pulse trains was found the most effective pattern for brain activity improvements, which supports our hypothesis.

As mentioned above, to evoke similar elbow flexion movements, wide-pulse stimulation for both CFT and VFT utilized lower current intensity than narrow-pulse stimulation suggesting that compared to NMES with narrow pulses, NMES with wide pulses had more advantages in activating muscle contraction. Wide-pulse pattern not only directly activated motor axons but also favored to depolarize the sensory fibers, resulting in recruiting motor units to evoke muscle contraction by the combination of the peripheral efferent with afferent pathways (Bergquist et al., 2011). The activated sensory fibers can generate the afferent volley ascending to the central nervous system (CNS) which contributes to the cortical excitability regulation, and the enhancement of cortical excitability is the key to the improvement of motor rehabilitation (Obayashi and Saito, 2022).

The present study focused on investigating sensorimotor cortical excitability induced by different NMES patterns through quantifying the beta ERD amplitudes of EEG signals at Electrode C3. Beta ERD amplitude has been reported to be strongly related to the level of sensorimotor cortical activation. The higher beta ERD amplitude is, the greater the cortical excitability is Tacchino et al. (2016). The results showed that compared to CFT patterns, VFT patterns favor the activation of sensorimotor cortical activities (i.e., significantly higher beta ERD amplitudes), which may be attributed to the way that VFT evokes muscle contraction. VFT was more prone to recruiting motor units in asynchronous pattern (i.e., more natural recruitment method) (Carole et al., 2016), and asynchronous motor units were generally recruited from the direct activation of motor axons (peripheral pathway) and the depolarization of sensory axons (central pathway) that activates motoneurons in the spinal cord (Bergquist et al., 2011). Together with those results in the present study, although CFT and VFT evoked similar elbow flexion movements, the stimulation current applied to right arm biceps of subjects in VFT pattern not only activated the motor axons, but also depolarized sensory fibers. This was also confirmed in the study of the MUs recruitment method during NMES-evoked muscle contraction (Collins, 2007). The sensory information generated by the activation of afferent fibers by electrical stimulation is transmitted to the central nervous system via the ascending afferent pathway. Siobhan’s research also demonstrated that afferent inputs generated by electrical stimulation through activating sensory axons could enhance cortical activity, driving transient or even long-term plastic changes to promote motor rehabilitation (Schabrun et al., 2012). Therefore, it may be speculated that VFT can induce larger beta ERD amplitudes due to the activation of more sensory axons.

Moreover, another important finding in this study was that the descending slope of beta ERD induced by VFT-evoked elbow flexion movements was significantly higher than that induced by CFT-evoked movements. This may be due to the fact that VFT activated more sensory axons and sensory volley ascending to the CNS for regulating cortical activities, thus developing the beta desynchronization more quickly. Previous study showed that electrical stimulation-evoked sensory inputs could regulate cortical activity networks (Carson and Buick, 2021). This was also verified in the monkey experiment that sensory afferents could encode cortical oscillations (Alonso et al., 2023). Therefore, the stimulation pattern of VFT in the present study was more inclined to depolarize sensory fibers so as to efficiently modulate cortical activities. The key to the improvement of motor function in motor rehabilitation lies in the enhancement of cortical excitability (Joy and Carmichael, 2021), and thus it is worthwhile to explore the potential of VFT in motor rehabilitation.

The results in the present study also showed that the pulse width of VFT could affect the sensorimotor cortical activities. We observed that wDFT induced higher amplitudes and the descending slope of beta ERD than nVFT did. By further comparison of the phase charges of the two VFT stimulation patterns, we found that to evoke similar elbow flexion motions, wDFT required significantly less phase charges than nVFT did. However, the beta ERD amplitude induced by the unit phase charge of wDFT was higher than that of nVFT, indicating that wDFT had higher phase charge efficiency to induce sensorimotor cortical excitability. In other words, the pulse assignment of VFT might regulate the level of cortical activities. Thus, the dual wide pulse train (wDFT) was more advantageous. The activation of axons is not only related to the accumulated charges applied to the membrane (Schwarz and Volkmer, 1965), but also affected by the physiological capacitance and input impedance of the membrane cells (Li and Bak, 1976). Due to the properties, such as large diameter, smaller input impedance and greater strength-duration constant, sensory fibers required less charges to be activated by the stimulation pulses with long sustained phase. Therefore, with longer pulse width (500 μs), wDFT in the present study required less phase charges, suggesting that more sensory axons could be activated by unit phase charge of wide-pulse VFT to better regulate cortical activities. Those results supported our hypothesis that VFT could induce stronger sensorimotor cortical excitability than CFT did. Further potential benefits on the modulation of cortical activities may be obtained by combining the wide-pulse with doublet VFT patterns.

The value of NMES in motor recovery has been widely reported. The activation of sensory fibers has become a new method to improve its clinical efficiency in rehabilitation due to the activation and regulation of central nervous activities by evoked sensory volley through afferent pathway (Obayashi and Saito, 2022). Wide-pulse stimulation has been shown to be more conducive to activating sensory fibers which have a larger intensity-time constant and lower base intensity than motor axons (Veale et al., 1973; Kiernan et al., 2004), but traditional CFT induced rapid muscle fatigue (Doucet et al., 2012). In contrast, VFT has been reported to induce fatigue-resistant muscle contraction and increase muscle contraction torque (Lagerquist and Collins, 2010; Karu et al., 1995). Some studies have suggested that this feature was due to the non-linear summation in induced muscle strength (Karu et al., 1995) and an intrinsic catch like property of muscle fibers itself (Stuart et al., 1991). Others have shown that motor units were recruited asynchronously during VFT-evoked muscle contraction (Carole et al., 2016). However, few studies have investigated whether VFT was more likely to activate sensory fibers when evoking muscle contraction. Therefore, the present study designed a new stimulation pattern combining wide pulse with VFT to investigate whether it may induce stronger cortical activities when evoking upper limb movements, thereby indirectly evaluate its activation of sensory axons. The results confirmed that wide-pulse VFT could not only induce stronger central nervous activities, but also establish the activation pattern more efficiently. In addition, although similar elbow flexion movements could be evoked by different stimulation patterns, both wide pulses and VFT enhanced sensorimotor cortical excitability by activating sensory fibers which is necessary for promoting motor rehabilitation. Therefore, those results in the present study further suggested that rehabilitation training should not be limited to joint movements, but also enhance sensory inputs ascending to sensorimotor areas.

Based on previous studies of VFT-evoked muscle contraction pattern and wide-pulse NMES for the activation of sensory fibers, the present study combined the two stimulation patterns to investigate whether VFT, especially the wide-pulse DFT, had more advantages of central cortical modulation over traditional CFT from the perspective of cortical activity regulation. The results showed that compared to simply increasing the pulse width in several previous studies, the comprehensive optimization of NMES patterns through the combination of wide pulse and VFT pattern was more conducive to enhancing cortical excitability. Therefore, the proposed NMES pattern may provide a new idea to optimize NMES parameters for the improvement of motor rehabilitation efficiency. However, the present study also has some limitations. The first one is the fixed order of four stimulation patterns applied in all subjects. This may cause a confounding impact from the stimulation order, such as muscle fatigue. Although every subject was provided a 10-min rest between any two NMES patterns to reduce muscle fatigue, it could be better to avoid this confounding factor using a randomized order of the four NMES patterns in different subjects. Early research in human brain electrophysiology has already investigated the cortical response to various kinematic parameters, and concluded that sensorimotor oscillation activities were different with different movements (Yuan et al., 2010; Cassim et al., 2000). Hence, further evaluations according to different kinematic characteristics should be designed in future for a more comprehensive understanding in the cortical modulation induced via dual wide pulse trains. Moreover, the participants involved in our study were all healthy subjects. Considering the reduction of movement-related beta ERD at the contralateral sensorimotor cortex in stroke patients (Wang et al., 2023), it is also necessary to extend the present findings more specific to pathological populations with motor function disorders in further studies.

## 5 Conclusion

This study investigated the effects of NMES with wide-pulsed DFT on sensorimotor cortical excitability. Our findings established that the induced cortical excitability could be improved by optimized pulse width and VFT pattern, whereby the dual 500 μs wide pulse train (wDFT) maximized the benefits. The profound reason may attribute to the facilitation of afferent fibers activation enhancement which consequently increased the ascending volleys. Therefore, our results implied the potential of wide-pulse DFT in assisting cortical excitability modulation through modifying the related afferent inputs, which suggested a promising usage in the improvement of rehabilitation efficiency for patients with motor dysfunction.

## Data Availability

The raw data supporting the conclusions of this article will be made available by the authors, without undue reservation.

## References

[ref1] Alonso I. Scheer I. Palacio-Manzano M. Frézel-Jacob N. Philippides A. Prsa M. (2023). Peripersonal encoding of forelimb proprioception in the mouse somatosensory cortex. Nat. Commun. 14:1866. doi: 10.1038/s41467-023-37575-w, PMID: 37045825 PMC10097678

[ref2] Backes W. H. Mess W. H. van Kranen-Mastenbroek V. Reulen J. P. H. (2000). Somatosensory cortex responses to median nerve stimulation: fMRI effects of current amplitude and selective attention. Clin. Neurophysiol. 111, 1738–1744. doi: 10.1016/S1388-2457(00)00420-X, PMID: 11018487

[ref3] Bergquist A. J. Clair J. M. Lagerquist O. Mang C. S. Okuma Y. Collins D. F. (2011). Neuromuscular electrical stimulation: implications of the electrically evoked sensory volley. Eur. J. Appl. Physiol. 111, 2409–2426. doi: 10.1007/s00421-011-2087-9, PMID: 21805156

[ref4] Carole C. Nicolas B. Gaëlle D. (2016). Effects of constant and doublet frequency electrical stimulation patterns on force production of knee extensor muscles. PLoS One 11:e0155429. doi: 10.1371/journal.pone.0155429 27167066 PMC4864221

[ref5] Carson R. G. Buick A. R. (2021). Neuromuscular electrical stimulation-promoted plasticity of the human brain. J. Physiol. 599, 2375–2399. doi: 10.1113/JP278298, PMID: 31495924

[ref6] Cassim F. Szurhaj W. Sediri H. Devos D. Bourriez J. L. Poirot I.. (2000). Brief and sustained movements: differences in event-related (de) synchronization (ERD/ERS) patterns. Clin. Off. J. Int. Federation Clin. Neurophysiol. 111, 2032–2039. doi: 10.1016/S1388-2457(00)00455-7, PMID: 11068239

[ref7] Collins D. F. (2007). Central contributions to contractions evoked by tetanic Neuromuscular electrical stimulation. Exerc. Sport Sci. Rev. 35, 102–109. doi: 10.1097/jes.0b013e3180a0321b, PMID: 17620928

[ref8] Deley G. Laroche D. Babault N. (2014). Effects of electrical stimulation pattern on quadriceps force production and fatigue. Muscle Nerve 49, 760–763. doi: 10.1002/mus.24210, PMID: 24639131

[ref9] Delorme A. Makeig S. (2004). EEGLAB: an open source toolbox for analysis of single-trial EEG dynamics including independent component analysis. J. Neurosci. Methods 134, 9–21. doi: 10.1016/j.jneumeth.2003.10.009, PMID: 15102499

[ref10] Doll B. D. Kirsch N. A. Bao X. Dicianno B. E. Sharma N. (2018). Dynamic optimization of stimulation frequency to reduce isometric muscle fatigue using a modified hill-Huxley model. Muscle Nerve 57, 634–641. doi: 10.1002/mus.25777, PMID: 28833237 PMC5817016

[ref11] Doucet B. M. Lam A. Griffin L. (2012). Neuromuscular electrical stimulation for skeletal muscle function. Yale J. Biol. Med. 85, 201–215. 22737049 PMC3375668

[ref12] Insausti-Delgado A. López-Larraz E. Omedes J. Ramos-Murguialday A. (2021). Intensity and dose of neuromuscular electrical stimulation influence sensorimotor cortical excitability. Front. Neurosci. 14:593360. doi: 10.3389/fnins.2020.593360, PMID: 33519355 PMC7845652

[ref13] Jiang S. L. Wang Z. Yi W. He F. Qi H. Ming D. (2019). Current change rate influences sensorimotor cortical excitability during neuromuscular electrical stimulation. Front. Hum. Neurosci. 13:152. doi: 10.3389/fnhum.2019.00152, PMID: 31156411 PMC6529745

[ref14] Joy M. T. Carmichael S. T. (2021). Encouraging an excitable brain state: mechanisms of brain repair in stroke. Nat. Rev. Neurosci. 22, 38–53. doi: 10.1038/s41583-020-00396-7, PMID: 33184469 PMC10625167

[ref15] Kampe K. K. W. Richard A. J. Dorothee P. A. (2000). Frequency dependence of the functional MRI response after electrical median nerve stimulation. Hum. Brain Mapp. 9, 106–114. doi: 10.1002/(sici)1097-0193(200002)9:2<106::aid-hbm5>3.0.co;2-y 10680767 PMC6871875

[ref16] Karamian B. A. Siegel N. Nourie B. Serruya M. D. Heary R. F. Harrop J. S.. (2022). The role of electrical stimulation for rehabilitation and regeneration after spinal cord injury. J. Orthop. Traumatol. 23:2. doi: 10.1186/s10195-021-00623-6, PMID: 34989884 PMC8738840

[ref17] Karu Z. Z. Durfee W. K. Barzilai A. M. (1995). Reducing muscle fatigue in FES applications by stimulating with N-let pulse trains. I.E.E.E. Trans. Biomed. Eng. 42, 809–817. doi: 10.1109/10.398642, PMID: 7642195

[ref18] Kiernan M. C. Lin C. S. Y. Burke D. (2004). Differences in activity-dependent hyperpolarization in human sensory and motor axons. J. Physiol. 558, 341–349. doi: 10.1113/jphysiol.2004.063966, PMID: 15146048 PMC1664913

[ref19] Kimberley T. J. Lewis S. M. Auerbach E. J. Dorsey L. L. Lojovich J. M. Carey J. R. (2004). Electrical stimulation driving functional improvements and cortical changes in subjects with stroke. Exp. Brain Res. 154, 450–460. doi: 10.1007/s00221-003-1695-y, PMID: 14618287

[ref20] Lagerquist O. Collins D. F. (2010). Influence of stimulus pulse width on M-waves, H-reflexes, and torque during tetanic low-intensity neuromuscular stimulation. Muscle Nerve 42, 886–893. doi: 10.1002/mus.21762, PMID: 20886511

[ref21] Li C. L. Bak A. (1976). Excitability characteristics of the A- and C-fibers in a peripheral nerve. Exp. Neurol. 50, 1–79. doi: 10.1016/0014-4886(76)90236-3 1248547

[ref22] Makii M. Rebecca R. Lucia Z. Stephane P. Davide C. Matteo C.. (2015). Effects of increasing neuromuscular electrical stimulation current intensity on cortical sensorimotor network activation: a time domain fNIRS study. PLoS One 10:e0131951. doi: 10.1371/journal.pone.0131951 26158464 PMC4497661

[ref24] Müller G. R. Neuper C. Rupp R. Keinrath C. Gerner H. J. Pfurtscheller G. (2003). Event-related beta EEG changes during wrist movements induced by functional electrical stimulation of forearm muscles in man. Neurosci. Lett. 340, 143–147. doi: 10.1016/S0304-3940(03)00019-3, PMID: 12668257

[ref25] Obayashi S. Saito H. (2022). Neuromuscular stimulation as an intervention tool for recovery from upper limb paresis after stroke and the neural basis. Appl. Sci. 12:810. doi: 10.3390/app12020810

[ref26] Panizza M. Nilsson J. Roth B. J. Basser P. J. Hallett M. (1992). Relevance of stimulus duration for activation of motor and sensory fibers: implications for the study of H-reflexes and magnetic stimulation. Electroencephalogr. Clin. Neurophysiol. 85, 22–29. doi: 10.1016/0168-5597(92)90097-U, PMID: 1371740

[ref27] Powell J. Pandyan A. D. Granat M. Cameron M. Stott D. J. (1999). Electrical stimulation of wrist extensors in poststroke hemiplegia. Stroke 30, 1384–1389. doi: 10.1161/01.str.30.7.1384, PMID: 10390311

[ref28] Qu W. Hou W. Zhao Y. Shu B. Chen L. Zheng X.. (2020). Burst-modulated wide-pulse neuromuscular electrical stimulation enhances H-reflex recruitment in rats. Muscle Nerve 61, 535–541. doi: 10.1002/mus.26812, PMID: 31950518

[ref29] Rongsawad K. Ratanapinunchai J. (2018). Effects of very high stimulation frequency and wide-pulse duration on stimulated force and fatigue of quadriceps in healthy participants. Ann. Rehabil. Med. 42, 250–259. doi: 10.5535/arm.2018.42.2.250, PMID: 29765878 PMC5940601

[ref30] Ryoki S. Shinichi K. Masaki N. Shota M. Sho K. Kei S.. (2017). Presence and absence of muscle contraction elicited by peripheral nerve electrical stimulation differentially modulate primary motor cortex excitability. Front. Hum. Neurosci. 11:146. doi: 10.3389/fnhum.2017.00146 28392766 PMC5364169

[ref31] Schabrun S. M. Ridding M. C. Galea M. P. Hodges P. W. Chipchase L. S. (2012). Primary sensory and motor cortex excitability are co-modulated in response to peripheral electrical nerve stimulation. PLoS One 7:e51298. doi: 10.1371/journal.pone.0051298, PMID: 23227260 PMC3515545

[ref32] Schwarz F. Volkmer H. (1965). On the summation of local potentials under stimulation of motor nerve fibers with electrical alternating pulses. Acta Biol. Med. Ger. 15, 283–301. 5888104

[ref33] Stuart A. Binder M. Charles B. B. (1991). Use of a catchlike property of human skeletal muscle to reduce fatigue. Muscle Nerve 14, 850–857. doi: 10.1002/mus.880140909 1922180

[ref34] Tacchino G. Gandolla M. Coelli S. Barbieri R. Pedrocchi S. Bianchi A. M.. (2016). EEG analysis during active and assisted repetitive movements: evidence for differences in neural engagement. IEEE Trans. Neural Syst. Rehab. Eng. A Pub. IEEE Eng. Med. Biol. Soc. 25, 761–771. doi: 10.1109/TNSRE.2016.2597157 27529874

[ref35] Tenberg S. Mueller S. Vogt L. Roth C. Happ K. Scherer M.. (2023). Comparative effectiveness of upper limb exercise interventions in individuals with stroke: a network meta-analysis. Stroke 54, 1839–1853. doi: 10.1161/STROKEAHA.123.043110, PMID: 37293804

[ref36] Veale J. L. Mark R. F. Rees S. (1973). Differential sensitivity of motor and sensory fibers in human ulnar nerve. J. Neurol. Neurosurg. Psychiatry 36, 75–86. doi: 10.1136/jnnp.36.1.75 4348037 PMC494280

[ref37] Wang J. Bi L. Fei W. (2023). EEG-based motor BCIs for upper limb movement: current techniques and future insights. IEEE Trans. Neural Syst. Rehabil. Eng. 31, 4413–4427. doi: 10.1109/TNSRE.2023.3330500, PMID: 37930905

[ref38] Wang Z. Liu Y. Huang S. Huang H. Wu W. Wang Y.. (2024). Enhancing ERD activation and functional connectivity via the sixth-finger motor imagery in stroke patients. IEEE Trans. Neural Syst. Rehabil. Eng. 32, 3902–3912. doi: 10.1109/TNSRE.2024.3486551, PMID: 39453797

[ref39] Wegrzyk J. Ranjeva J. P. Fouré A. Kavounoudias A. Vilmen C. Mattei J.-P.. (2017). Specific brain activation patterns associated with two neuromuscular electrical stimulation protocols. Sci. Rep. 7:2742. doi: 10.1038/s41598-017-03188-9, PMID: 28577338 PMC5457446

[ref40] Yu P. Dong R. Wang X. Tang Y. Liu Y. Wang C.. (2024). Neuroimaging of motor recovery after ischemic stroke − functional reorganization of motor network. NeuroImage: Clinical 43:103636. doi: 10.1016/j.nicl.2024.103636, PMID: 38950504 PMC11267109

[ref41] Yuan H. Perdoni C. He B. (2010). Relationship between speed and EEG activity during imagined and executed hand movements. J. Neural Eng. 7:026001. doi: 10.1088/1741-2560/7/2/026001, PMID: 20168002 PMC3036745

[ref42] Zhang L. Chen L. Wang Z. Liu X. Ming D. (2023). Enhancing motor imagery performance by antiphasic 10 hz transcranial alternating current stimulation. IEEE Trans. Neural Syst. Rehabil. Eng. 31, 2747–2757. doi: 10.1109/TNSRE.2023.3286419, PMID: 37318971

[ref43] Zhao Y. Lai J. J. Wu X. Y. Qu W. Wang M. Q. Chen L. (2018). “Neuromuscular electrical stimulation with kilohertz frequency alternating current to enhance sensorimotor cortical excitability,” in 2018 40th Annual International Conference of the IEEE Engineering in Medicine and Biology Society (EMBC), 2240–2243. doi: 10.1109/EMBC.2018.8512855 30440851

